# Effects of Fermented Liquid Feed with Compound Probiotics on Growth Performance, Meat Quality, and Fecal Microbiota of Growing Pigs

**DOI:** 10.3390/ani15050733

**Published:** 2025-03-04

**Authors:** Mengting Ji, Xiaoyin Rong, Yifan Wu, Haonan Li, Xiaolei Zhao, Yan Zhao, Xiaohong Guo, Guoqing Cao, Yang Yang, Bugao Li

**Affiliations:** Key Laboratory of Animal Science, College of Animal Science, Shanxi Agricultural University, No.1, Mingxian South Road, Taigu, Jinzhong 030801, China; jimengting2864@163.com (M.J.); z20203359@stu.sxau.edu.cn (Y.W.); zhaoyan@sxau.edu (Y.Z.); xhguo@sxau.edu.cn (X.G.); sxndcgq@sxau.edu.cn (G.C.)

**Keywords:** fermented liquid feed, growing pigs, meat quality, growth performance, gut microorganism

## Abstract

Feed fermentation enhances feed nutrition and animal health, but its impact on the gut microbiota of young pigs remains unclear. This study aimed to evaluate the effects of a probiotic fermented feed, which includes *Lactobacillus plantarum*, *Pediococcus pentosaceus*, *Bacillus subtilis*, and *Bacillus coagulans*, on the growth performance, meat quality, and intestinal microbiota of growing pigs. We randomly assigned 24 Duroc × Landrace × Landrace pigs to two groups: a control (Ctrl) group and the fermented liquid feed (FLF) group, with three replicate pens per group and four pigs per pen. Results indicated that the FLF group experienced a significant decrease in anti-nutritional factors like α-conglycinin and β-conglycinin. In addition, the average daily gain of pigs in the FLF group increased significantly, while the feed conversion ratio and shear force decreased. HE staining showed that the FLF group had notably enhanced villus height in the jejunum and ileum. 16S rRNA sequencing revealed a marked increase in the relative abundance of certain microbes in the FLF group, which were predominantly associated with carbohydrate and amino acid metabolism. These results indicated that compound probiotic FLF can elevate feed quality, enhance the growth performance of growing pigs, and ameliorate the structure of the gut microbiota.

## Simple Summary

Fermentation of feed is key to boosting the nutritional value of feedstuffs, improving the health of livestock and poultry. However, the effects of probiotic fermented feed on the gastrointestinal microbiota of growing pigs are not completely understood. To better understand the effects of a liquid probiotic feed, we concurrently examined the pigs fed with a basal diet and the fermented liquid feed (FLF). The results indicate that using a compound probiotic fermented liquid feed improves feed quality, enhances the growth performance of growing pigs, and ameliorates the gut microbiota, consequently leading to improved morphology of the small intestine and the maintenance of intestinal health.

## 1. Introduction

Corn and soybean meal, as staple components of swine diets, provide essential energy, protein, and nutrients [1]. Yet, these feeds contain anti-nutritive elements and factors that potentially impair pig health and growth performance [2,3]. Particularly, anti-nutritional factors (ANFs) in soybean meal impede nutrient digestion and absorption [4], and may induce inflammation and oxidative stress, often due to gastrointestinal dysbiosis and an immature immune system, leading to compromised intestinal function [5,6]. Thus, this study aims to explore whether multi-strain synergistic fermentation can effectively reduce anti-nutritional factors in corn–soy ration, thereby enhancing feed quality and optimizing nutrient utilization, with the objective of improving the health and growth of pigs.

Microbial fermentation, particularly through fermented liquid feed (FLF), is an advanced feed processing technique. Extensive research confirms the benefits of FLF in reducing weaning stress and improving nutrient bioavailability and nutritional value in piglets [7,8]. FLF has also been shown to markedly improve feed efficiency, intake, carcass weight gain, and overall growth performance [9,10]. Additionally, FLF is associated with enhanced gastrointestinal health in piglets [11]. Liquid fermented feeds typically contain single or compound probiotic cultures, with the latter demonstrating more pronounced synergistic effects [12,13]. Consequently, this study innovatively developed a composite fermented liquid feed made up of *Lactobacillus plantarum*, *Pediococcus pentosaceus*, *Bacillus subtilis*, and *Bacillus coagulans*, where the lactobacillus notably acidifies feed by producing lactic acid and lowering pH, while Bacillus augments Lactobacillus activity. Research indicates that Bacillus inclusion in anaerobic fermentation swiftly exhausts residual oxygen and boosts lactic acid bacteria’s acid production in subsequent stages [14].

The gut microbiota is pivotal for nutrient absorption, metabolism, and immune system functioning [15]. The underdeveloped intestines of pigs are susceptible to microbial invasions, which can compromise the integrity of the intestinal barrier and disrupt the ecological equilibrium of the gut microbiota [16]. While studies have demonstrated that FLF enhances intestinal microbial balance in pigs [17], the effects of various probiotic fermentation diets and supplementation levels on the gastrointestinal flora’s structure, abundance, diversity, and functionality in growing pigs are not yet fully understood. Thus, the objective of this study is to assess a novel probiotic formula and its application, aiming to provide a scientifically validated liquid fermentation bacteria formula for practical production use.

## 2. Materials and Methods

### 2.1. Materials

Standard gluconic acid was purchased from Solarbio (Beijing, China). Total amino acid assay kits (A026-1-1) were purchased by Jiancheng Bioengineering Technology (Nanjing, China). ELISA kits for detecting glycinin, α-conglycinin (α-CY), β-conglycinin (β-CY), and soybean trypsin inhibitor (STI) content were purchased from Jiangsu Meimian Industrial Co., Ltd. (Yancheng, China). All chemicals and solvents used were of analytical grade.

### 2.2. Preparation of Fermented Liquid Feed (FLF)

The basal diet was formulated according to the nutritional requirements recommended by the National Research Council [18], with the composition and nutritional value shown in Table 1. The substrate for the fermented liquid feed (FLF) consists of a solid basal diet, and the preparation method is as follows: First, activate *Lactobacillus plantarum*, *Bacillus subtilis*, *Pediococcus pentosaceus*, and *Bacillus coagulans*, and cultivate them in liquid culture medium for 24 h, mixing the four bacterial suspensions in a 1:1:1:1 ratio. Then, inoculate the mixed bacterial suspension into a mixture of feed and water (1:2.5) at an inoculation rate of 6%, place it in a sterile sealed bag, and ferment it in an electric heating incubator at 30 °C for 3–5 days. Each experiment was conducted in triplicate.

### 2.3. Feeding-Experiment Design

Twenty-four healthy Duroc × Landrace × Yorkshire pigs, aged 75 ± 3 days old and weighing 27.97 ± 4.48 kg, were randomly divided into a control (Ctrl) group and a fermented liquid feed (FLF) group. Each group had three replicates with four pigs (two males and two females). The Ctrl group was fed a standard basal diet, while the FLF group was fed a mixture of 10% fermented liquid feed and 90% basal diet. During the experiment, feed was provided twice daily in the morning and evening, and the remaining feed in the troughs was measured. Additionally, the temperature, humidity, and odor in the pigpens were monitored daily, with ventilation as needed. The pre-feeding period was 5 days, followed by a 30-day formal trial, after which the pigs were slaughtered. Before the start of the experiment, deworming, castration, and vaccination were carried out according to standard farm protocols.

### 2.4. Samples Collection

Feed intake was recorded after feeding each morning and evening. At the end of the trial, three pigs close to the average body weight were selected from each group for jugular blood sampling. After slaughter, portions of the ileum and colon tissues were preserved in 4% paraformaldehyde solution, and the remaining tissues were quickly frozen in liquid nitrogen and stored in a −80 °C freezer for subsequent analysis.

### 2.5. Determination of Nutrient Digestibility

Using the total feces collection method, one pig per replicate was randomly selected and raised in a metabolic cage. On day 14 of the trial, fresh fecal samples were collected and treated with 10% sulfuric acid for nitrogen fixation (10 mL of sulfuric acid per 100 g of feces), then mixed and stored at −20 °C in a freezer. After drying the feces, relevant indices were measured, including organic matter (OM), crude protein (CP), ether extract (EE), neutral detergent fiber (NDF), and acid detergent fiber (ADF), which were determined according to established methods [19].

### 2.6. Meat Quality Analysis

Pork quality includes measurements such as pH, meat color, and shear force. To determine drip loss, the longissimus dorsi muscle from the third and fourth last ribs is weighed 1 to 2 h post-slaughter. Meat color is assessed using a spectrophotometer (Konica Minolta, Tokyo, Japan) on the longissimus dorsi muscle at the thoracolumbar junction. For cooking loss, the psoas major muscle from the left side of the carcass is placed in an aluminum pot and steamed in boiling water for 30 min. After steaming, it is hung in a cool place for 30 min before weighing and recording the cooking loss. Immediately after the pig stops breathing, the pH of the longissimus dorsi muscle from the first to second last ribs is measured using a pH meter (pH-STAR, SFK-Technology, Herlev, Denmark) within 45 min (recorded as pH_45min_) and again at 24 h (recorded as pH_24h_). Shear force is evaluated using the Warner–Bratzler shear test method 24 h after sample collection.

### 2.7. Determination of Volatile Fatty Acids (VFAs)

Following the methods established by previous researchers [20], volatile fatty acids were identified using gas chromatography (TRACE 1300 Gas Chromatograph Mass Spectrometer, Thermo Scientific, Waltham, MA, USA). For this analysis, mix 0.5 g of the fermentation feed supernatant with 2 mL of deionized water and centrifuge at 12,000 rpm. Then, take 0.5 mL of the supernatant and mix it with 0.1 mL of crotonic acid metaphosphate solution, and store the mixture at −20 °C. After overnight thawing, filter the sample through a 0.22 μm water membrane, centrifuge, and then inject 1 μL of the supernatant into the gas chromatograph for detection. The chromatographic conditions were set according to the team’s previous settings [21]: injection temperature of 220 °C, split ratio 6, constant flow rate of 0.8 mL/min, initial column temperature of 70 °C, and detector temperature of 220 °C. Gas flow rates were set to hydrogen 35 mL/min, air 350 mL/min, and tail blow 40 mL/min.

### 2.8. Circle of Inhibition Test

The intestinal pathogens *Escherichia coli* and *Salmonella* were activated and prepared into bacterial suspensions, with the OD value controlled between 0.3 and 0.36. Subsequently, 8 mL of the suspension was added to 400 mL of solid culture medium, mixed thoroughly, and poured into sterile Petri dishes to solidify. Then, two Oxford cups were symmetrically placed on the medium, one cup filled with 100 μL of sterile water as a control, and the other cup filled with 100 μL of the fermentation liquid. The dishes were incubated in a 37 °C incubator for 24 h, after which the sizes of the inhibition zones were measured. Each treatment was repeated three times.

### 2.9. Histological Evaluation

Jejunum and ileum samples fixed in 4% paraformaldehyde (Solarbio Life Sciences, Beijing, China) were dehydrated, cleared, and embedded in paraffin. Tissue sections were cut at a thickness of 5 μm using a rotary microtome (Leica, Wetzlar, Germany) according to a previously reported protocol [22], stained with hematoxylin and eosin, and imaged using the EVOSFL (Life Technologies, Carlsbad, CA, USA) automated cell imaging system. Morphometric parameters, including villus height (VH), crypt depth (CD), and the ratio of villus height to crypt depth (V/C), were determined using Image-Pro Plus 6.0 software.

### 2.10. 16S rRNA Sequencing

Starting from the 4th week, fresh fecal samples were collected daily in the morning from the anus of each pig in the pen. These samples were promptly immersed in liquid nitrogen and subsequently stored in a −80 °C freezer for preservation.

To extract microbial DNA from pig feces, we utilized the Hipure Fecal DNA Kit (Magen, Guangzhou, China), followed by PCR amplification targeting the 16S rRNA gene. Post-amplification, we employed the AMPure XP Kit (Beckman, Brea, CA, USA) for purification of the PCR products. Sequencing of the 16S amplicons was conducted on the Illumina HiSeq 2500 platform. Raw fastq data underwent quality filtering using FASTP (version 0.18.0), and operational taxonomic units (OTUs) were clustered at a 97% similarity level with UPARSE (version 9.2.64). Chimeric sequences were identified and removed using the UCHIME algorithm. Taxonomic annotation was performed with the RDP classifier (version 2.2) against the UNITE database (version 8.0), with a confidence threshold of 0.8. At Megi Biotech, 16S rRNA sequencing and data analysis were conducted using the sklearn algorithm to classify Amplicon Sequence Variant (ASV) species. The microbial alpha diversity in fecal samples was evaluated with four indices: Chao, Shannon, Simpson, and Faith, with significant inter-group differences in these alpha diversity indices being compared. Post-screening, the characteristic phyla and bacteria with the highest total abundance across different groups were identified. Endemic and shared characteristic bacteria and genera were determined for each sample group using the provided abundance data. Biological markers with significant inter-group differences were pinpointed, and a linear discriminant analysis effect size (LEfSe) map was constructed. Metagenomic predictions were carried out using the ASV abundance table and ASV sequences to analyze the metabolic functional pathways of specific genes via Kyoto Encyclopedia of Genes and Genomes (KEGG) functional prediction analysis. Correlations between bacterial abundance and volatile fatty acids in the two groups were further explored and analyzed in light of the volatile fatty acid content findings.

### 2.11. Statistical Analysis

In this experiment, data were processed using SPSS 20.0. Independent samples *t*-test was used to analyze the data, and the results were expressed as “mean ± SEM”. Statistical significance was indicated by a *p* value of less than 0.05.

## 3. Results

### 3.1. Nutrient Composition of Compound Probiotic Fermented Liquid Feed

The nutrient composition of probiotic complex fermented diets is shown in Table 2. The results showed that the contents of CP, organic matter, and total amino acids in the FLF group were significantly higher than those in the Ctrl group (*p* < 0.01).

### 3.2. Effect of Compound Probiotic Fermented Liquid Feed on Anti-Nutritional Factors, Intestinal Pathogenic Bacteria, and Volatile Fatty Acids

**Figure 1:**
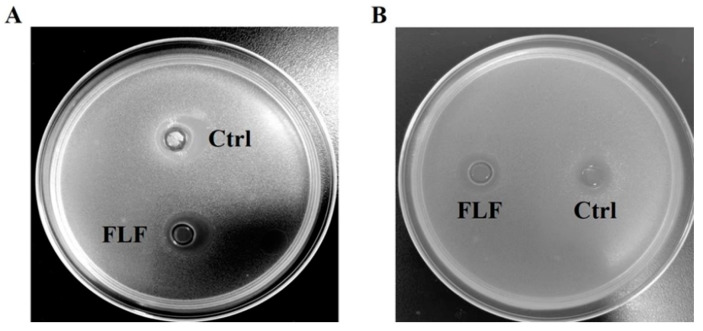
The antimicrobial effect of the fermentation liquid on harmful pathogens: (**A**) is *Escherichia coli* plate; (**B**) is *Salmonella* plate.

The antibacterial experiments demonstrated that the inhibition zone diameter in the FLF group was significantly larger than in the Ctrl group for both *E. coli* and *Salmonella* plates (*p* < 0.01) (Figure 1, Table 3), signifying the fermentation broth’s inhibitory impact on these pathogens. Additionally, ELISA assays revealed that the FLF group exhibited a significant reduction in the levels of four anti-nutritional factors compared to the Ctrl group (*p* < 0.01), with α-CY decreasing by 17.07%, β-CY by 17.45%, Globulin by 32.04%, and STI by 15.42% (Table 4). Moreover, as presented in Table 5, the diet resulted in a lowered pH due to fermentation and generated a range of volatile fatty acids, including lactic acid, acetic acid, isobutyric acid, and pentanoic acid.

### 3.3. Effect of Fermented Liquid Feed on Phenotype of Growing Pigs

Phenotypic data from growing pigs were utilized to assess the effect of liquid fermented probiotic feed on their growth performance. The findings indicated that, compared to the Ctrl group, the FLF group showed an increase in ADG by 9.17%, while F/G decreased by 11.69% (Table 6). In addition, the FLF group exhibited a reduction in GLU levels by 3.27%, TC by 4.98%, and INS by 14.27%. In contrast, the TP levels in the FLF group increased by 5.36%, and ALB levels increased by 6.95% (Table 7).

### 3.4. Effect of Fermented Liquid Feed on Pig Nutrient Digestibility and Meat Quality

The study investigated the effects of FLF on the apparent digestibility in pigs, and the results showed that while the total apparent digestibility did not change significantly, the digestibility of CP in the FLF group increased by 10.84% (Table 8). In addition, FLF significantly influenced the meat quality parameter. Specifically, the FLF group exhibited a reduction in drip loss by 34.44%, cooking loss by 19.43%, and yellowness of meat color (b*) by 13.31%, while redness of meat color (a*) increased by 8.95% (Table 9).

### 3.5. Effect of Fermented Liquid Feed on Pig Intestine

**Figure 2:**
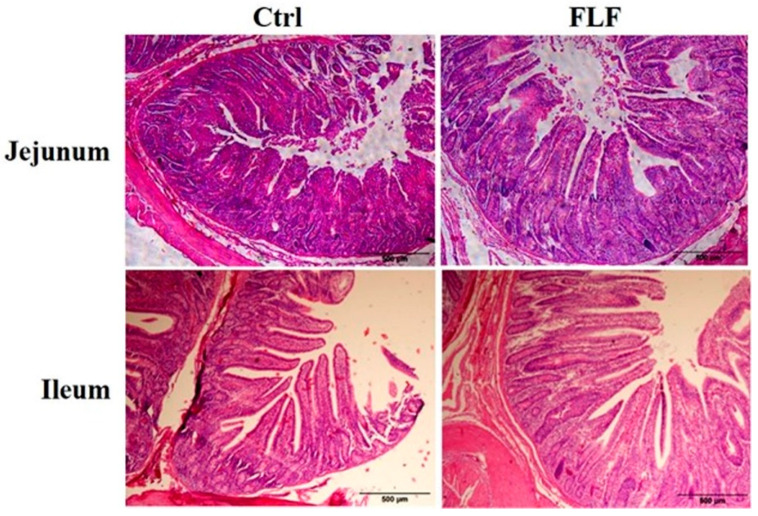
Effects of fermented liquid feed on the morphological structures of the jejunum and ileum (100×).

Morphological alterations in the jejunum and ileum were assessed through HE staining (Figure 2). The results indicated that FLF significantly elevated the villus height and crypt depth in the jejunum (*p* < 0.01), with the villus height increasing by 64.56% and the crypt depth by 60.41%. Additionally, the FLF group showed an increase in villus height by 27.91%, while the villus height-to-crypt depth ratio in the ileum was significantly higher in the FLF group than in the Ctrl group (*p* < 0.01) (Table 10). These results suggest that FLF can improve the morphological structure of the small intestine, particularly in the jejunum and ileum, which in turn may bolster intestinal digestion and absorption efficiency.

### 3.6. Fecal Microbial Diversity and Composition Differences

**Figure 3:**
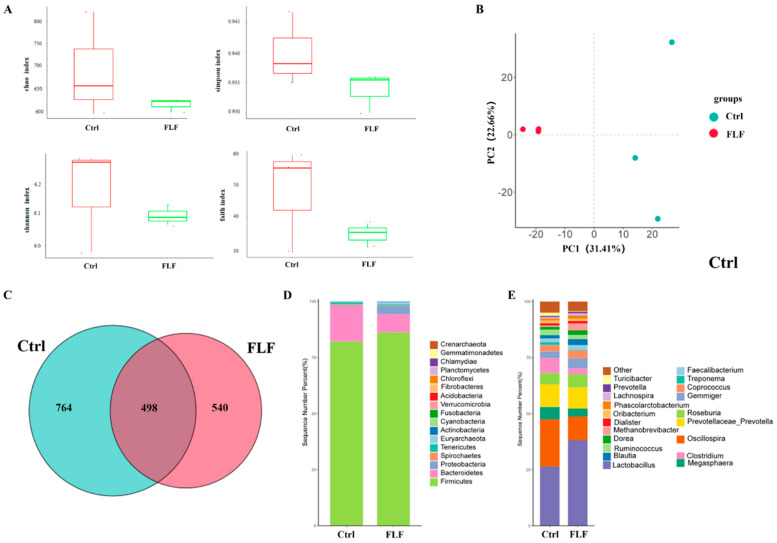
Effect of compound probiotic feed on the structure of fecal microbial flora in growing pigs. (**A**) Alpha diversity index of fecal microbiota; (**B**) results of principal components analysis of fecal microbiota; (**C**) Venn diagram of fecal microbiota; (**D**) phylum-level abundance; (**E**) genus-level abundance.

Fecal microbial composition was determined through 16S rRNA sequencing, with findings presented in Figure 3. Alpha diversity analysis revealed that the Faith phylogenetic diversity index for the FLF group was significantly lower than that of the Ctrl group (Figure 3A), suggesting a decrease in microbial diversity following fermentation. Principal component analysis (PCA) depicted a distinct divergence in community structure between the FLF group and the Ctrl group (Figure 3B), and the operational taxonomic unit count in the FLF group was modestly lower than in the Ctrl group (Figure 3C). Further taxonomic abundance analysis identified over seven bacterial phyla, with Firmicutes as the predominant phylum, succeeded by Bacteroidetes (Figure 3D). At the genus level, more than 20 bacterial genera were detected, and among these, Lactobacillus, Prevotella, Blautia, and Gemiger were notably more abundant in the FLF group compared to the Ctrl group (*p* < 0.01) (Figure 3E).

### 3.7. Functional Prediction and Correlation Analysis of Fecal Microbiota

**Figure 4:**
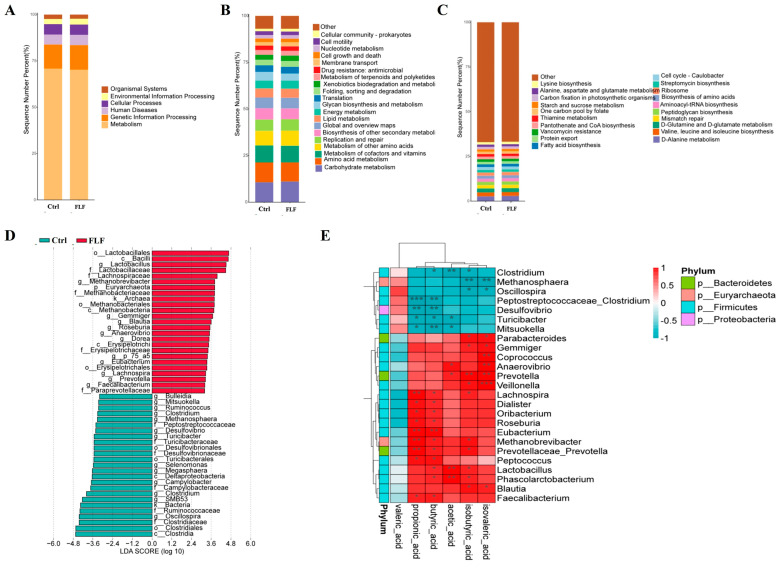
KEGG enrichment analysis of fecal microbiome and heatmap of microbial and environmental factors. (**A**–**C**) KEGG pathways I to III; (**D**) linear discriminant analysis effect size diagram of fecal microbiota, whereby c represents class, o represents order, f represents family, and g represents genus; (**E**) heatmap showing correlations, with red indicating positive correlation and blue indicating negative correlation. * *p* < 0.05, ** *p* < 0.01 and *** *p* < 0.001.

The PICRUSt2 software (version 2.1.4) was employed to forecast KEGG pathway functions, with outcomes depicted in Figure 4A–C. The analysis indicated a predominant enrichment of these genes in six distinct pathways, encompassing metabolism, genetic information processing, and biological systems. The relative gene abundance in carbohydrate metabolism and amino acid metabolism was significantly higher in the FLF group compared to the Ctrl group (*p* < 0.01). LEfSe analysis pinpointed Lactobacillales, Bacilli, and Lactobacillus as the most significant strains in the FLF group, in contrast to Clostridia, Clostridiales, and Ruminococcaceae in the Ctrl group (Figure 4D). Spearmen’s correlation coefficient was utilized to assess the correlations between the top 25 bacterial species and six environmental factors (Figure 4E). Over 15 bacterial species exhibited positive correlations with volatile fatty acids. Notably, Lactobacillus, Phascolarctobacterium, Prevotella, and acetic acid demonstrated significant positive correlations, whereas Clostridium was negatively correlated with acetic acid. Methanobrevibacterium, Rotella, Lachnospira, and Prevotella were significantly positively correlated with propionic acid. Butyric acid showed significant positive correlations with Eubacterium (*p* < 0.01) and also with Microbacterium and Lactobacillus (*p* < 0.05). Further examination of intestinal volatile fatty acids revealed that the FLF group had considerably higher levels than the Ctrl group (*p* < 0.05). Isobutyric acid and isovaleric acids were significantly elevated in the FLF group (*p* < 0.01), while valeric acid displayed no significant variation (Table 11).

## 4. Discussion

Soybean meal and corn are crucial to animal husbandry due to their high protein and energy content. This research indicates that FLF significantly increases the CP content in feed (*p* < 0.01), which is attributed to the bacterial growth process releasing bacterial proteins that can serve as a direct protein source [23]. Consistent with Shi et al. [24], the significant increase in total amino acid content suggests that proteins are degraded into more digestible smaller molecules. Additionally, the difference in the increase between CP and total amino acids content may be related to the probiotic in the fermentation process enhancing nitrogen utilization, specific amino acid synthesis distribution, and the formation of small peptides [25,26]. Although anti-nutritional factors in feed such as protease inhibitors and monensin can reduce nutrient absorption, the FLF effectively degrades soybean globulin, α-CY and β-CY, and STI through the fermentation process. Its efficiency, though lower than that reported by Zheng et al. [27], confirms the potential for multi-factor synergistic degradation. Additionally, the pH value of FLF dropped to 4.38, reaching the ideal value evaluated by Van (pH < 4.5) [28], while detecting lactic and acetic acids, but not propionic and butyric acids, possibly due to bacterial strain metabolic preferences and process design differences. Moreover, FLF’s inhibition rates against *E. coli* and *Salmonella* were significant, likely due to the high concentration of lactic acid and VFAs’ antibacterial effects and the acidic environment inhibiting the proliferation of pathogens. Therefore, this study introduces a new probiotic formula and its application, providing a foundation and reference for further research on fermented feed.

Growth performance is a core indicator for assessing the efficiency of the swine industry and animal welfare. This study demonstrates that FLF significantly increased the average daily gain of growing pigs and reduced the feed conversion ratio. The mechanism may be due to the sour and fragrant flavor of the fermented feed and its good palatability, as well as the enhanced gastric acidity that stimulates appetite. Interestingly, there was no significant difference in final weight between the FLF group and the Ctrl group, although there was a trend of increase, which may be due to differences in physical condition or too short a feeding period. Serum cholesterol reflects the absorption and metabolism of lipids in the animal’s body [29]. In this experiment, an increase in total protein and a decrease in total cholesterol in the FLF group indicates that FLF enhanced the capacity for protein synthesis metabolism in the blood. Although there was no significant overall difference in apparent digestibility in this experiment, the nutrient digestibility of CP increased by 10.84%, which may be related to the efficient absorption of microbial protein and the removal of anti-nutritional factors that reduce the burden on the gut. To assess the potential impact of the feed formulation on changes in meat quality, we measured the color of the meat, with a higher redness value typically indicating better meat quality [2,30]. Meat quality analysis showed that the redness of the FLF group was significantly higher than that of the Ctrl group, with significant reductions in drip loss and shear force, indicating that the use of liquid fermented feed improved the meat quality and tenderness of growing pigs and reduced water loss [31]. Additionally, FLF significantly increased the villus height of the jejunum and the villus/crypt ratio of the ileum in growing pigs (*p* < 0.01), consistent with the results reported by Lin et al., [32], indicating that FLF can improve the morphology of the small intestine, especially the jejunum and ileum, and promote the intestinal digestion and absorption of pigs.

The gut microbiota plays a pivotal role in metabolism, nutrient absorption, and immune function [33]. It is capable of secreting a range of digestive enzymes that facilitate the breakdown of organic matter in feed. Probiotics, when integrated into animal feed in optimal quantities, can alter the gut microbiota composition, mitigate intestinal disorders, strengthen the immune system, and enhance overall gut health and productivity in swine [34,35]. There are more than 500 kinds of microorganisms in the pig’s gut, with *Firmicutes, Bacteroidetes*, and *Proteobacteria* being the most dominant. 16S rRNA sequencing revealed that after the intervention with FLF, *Firmicutes*, *Bacteroidetes*, and *Lactobacillus* were significantly enriched, indicating that the *Lactobacillus plantarum* and *Pediococcus pentosaceus* in the fermented feed may have a good colonization effect in the gut. Extensive research highlights the essential role of VFAs, such as acetate, butyrate, and propionate, in maintaining metabolic balance and overall gut health in humans and animals [36,37]. Meanwhile, VFAs regulate intestinal motility and hormone secretion by binding to G protein-coupled receptors on intestinal epithelial cells [38]. In this experiment, the levels of acetic acid, propionic acid, and butyric acid in the colon of the FLF group were significantly increased, indicating that liquid fermented feed increased the concentration of VFAs in the gut. Further KEGG enrichment analysis found that the FLF increased the abundance of genes related to carbohydrate metabolism and some amino acid metabolism pathways. In addition, the amino acid metabolic pathway activity was enhanced after the intervention with FLF, promoting the efficient utilization of carbon sources by the microbiota community. The results above indicate that after the liquid fermented feed enters the pig intestine, the abundance of relevant probiotic groups significantly increases. During this process, diverse volatile fatty acids are biosynthesized, which have the function of inhibiting pathogenic bacteria, and are conducive to maintaining the balance of the microbial community in growing pigs.

## 5. Conclusions

The nutritional value of the compound probiotic fermented feed was significantly greater than that of the basal diet. This diet not only reduced anti-nutritional factors and curbed the proliferation of intestinal pathogens but also led to a substantial increase in the average daily weight gain of growing pigs. It enhanced meat quality, boosted the digestibility of crude protein, and positively impacted the morphology of the jejunum and ileum. Additionally, it optimized the structure of the intestinal flora, augmented various metabolic pathways, and promoted intestinal digestion and absorption, thereby preserving the overall intestinal health of growing pigs. This feed could be used in future animal nutrition strategies to provide a sustainable and effective solution for the swine industry.

## Figures and Tables

**Table 1:** Composition and nutrient content of feed.

Items	Contents (%)	Nutrient Levels ^(2)^	Contents (%)
Corn	61.73	DM	87.17
Expanded soybean	20.87	CP	18.00
Soybean	11.02	EE	6.02
Lysine hydrochloride	0.50	CA	0.65
Met	0.22	CF	2.39
Thr	0.20	Lys	1.32
Trp	0.08	Met	0.49
Limestone	0.34	Met + Cys	0.79
CaHPO_4_	2.07	Thr	0.86
NaCl	0.67	Trp	0.27
Acidifier	0.30		
Premix ^(1)^	2.00		
Total	100.00		

CP = crude protein; EE = ether extract; CA = Crude Ash; CF = Crude Fiber; Met = Methionine; Thr = Threonine; Trp = Tryptophan. ^(1)^ The premix diet was provided with the following additives per kg diet: vitamin C, 1.5 mg, vitamin A, 9000 IU; vitamin B1, 5 mg; vitamin B2, 9 mg; vitamin B6, 32 mg; vitamin B12, 0.030 mg; vitamin D3, 1900 IU; vitamin E, 22 IU; vitamin K, 35 mg; biotin 0.09 mg; calcium pantothenate, 15 mg; niacin, 29 mg; Cu, 21 mg; Fe, 65 mg; Mn, 45 mg; Zn, 65 mg; I, 0.55 mg; Se, 0.32 mg. ^(2)^ Nutrient levels were calculated.

**Table 2:** Quality of compound probiotics fermented liquid feed.

Items	Ctrl	FLF
Water, %	13.90 ± 0.08	8.58 ± 0.41 **
Ash, %	6.09 ± 0.02	6.12 ± 0.15
Organic matter, %	80.01 ± 0.09	85.30 ± 0.55 **
CP, %	18.00 ± 0.04	25.98 ± 0.18 **
EE, %	15.56 ± 0.17	15.03 ± 0.74
NDF, %	11.19 ± 0.08	11.05 ± 0.55
ADF, %	4.43 ± 0.03	4.27 ± 0.36
Total amino acids, g/100 g	6.80 ± 0.07	8.43 ± 0.23 **

CP = crude protein; EE = ether extract; NDF = neutral detergent fiber; ADF = acid detergent fiber. For the same row, statistically significant differences between Ctrl and FLF groups were considered: ** *p* < 0.01. Data are expressed as means ± SEM (n = 3).

**Table 3:** Inhibition zone diameters of fermentation broth against harmful pathogens.

Indicator Bacteria	Ctrl	FLF
*E. coli* (mm)	7.8 ± 0.0	15.7 ± 0.3 **
*Salmonella* (mm)	7.8 ± 0.0	12.3 ± 0.2 **

*E. coli* = *Escherichia coli.* For the same row, statistically significant differences between Ctrl and FLF groups were considered: ** *p* < 0.01. Data are expressed as means ± SEM (n = 3).

**Table 4:** Effects of fermented liquid feed on the content of anti-nutritional factors in feed.

Anti-Nutritional Factor	Ctrl	FLF
α-CY (μg/mL)	96.14 ± 0.94	79.72 ± 0.78 **
β-CY (μg/mL)	69.95 ± 1.38	57.94 ± 0.63 **
Globulin (μg/mL)	195.07 ± 2.80	132.75 ± 1.33 **
STI (μg/mL)	454.06 ± 2.62	384.06 ± 3.32 **

α-CY = α-conglycinin; β-CY = β-conglycinin; STI = soybean trypsin inhibitor. For the same row, statistically significant differences between Ctrl and FLF groups were considered: ** *p* < 0.01. Data are expressed as means ± SEM (n = 3).

**Table 5:** The pH value and VFA content of the liquid fermented feed.

Items	FLF
pH	4.38 ± 0.01
Lactic acid (mmol/L)	96.94 ± 0.02
Acetic acid (mmol/L)	4.28 ± 0.67
Propionic acid (mmol/L)	-
Butyric acid (mmol/L)	-
Isobutyric acid (mmol/L)	0.06 ± 0.01
Pentanoic acid (mmol/L)	0.10 ± 0.07
Total volatile fatty acid concentration (mmol/L)	101.38

“-” indicates not detected; none of the volatile fatty acids were detected in the Ctrl group.

**Table 6:** Effects of fermented liquid feed on growth performance of growing pigs.

Items	Ctrl	FLF
First weight (kg)	27.72 ± 1.86	28.22 ± 2.36
Final weight (kg)	45.59 ± 3.17	47.59 ±4.40
ADG (g)	658.72 ±12.06	719.00 ± 16.04 *
ADFI (g)	1812.66 ± 29.57	1751.07 ± 62.62
F/G	2.75 ± 0.01	2.43 ± 0.03 **

ADG = average daily gain; ADFI = average daily feed intake; F/G = feed/gain. For the same row, statistically significant differences between Ctrl and FLF groups were considered: * *p* < 0.05 and ** *p* < 0.01. Data are expressed as means ± SEM (n = 3).

**Table 7:** Effects of fermented liquid feed on the serum biochemical indices of growing pigs.

Indicator Bacteria	Ctrl	FLF
UN (mmol/L)	4.52 ± 0.32	5.09 ± 0.19
GLU (mmol/L)	6.73 ± 0.08	6.51 ± 0.07 *
TP (g/L)	68.03 ± 0.99	71.67 ± 0.72 **
ALB (g/L)	36.57 ± 1.2	39.11 ± 0.74 *
GLO (g/L)	31.47 ± 0.75	32.56 ± 0.02
TC (mmol/L)	2.81 ± 0.07	2.67 ± 0.04 *
ALP (U/L)	174.34 ± 2.33	180.33 ± 1.76
GH (µg/L)	20.14 ± 1.30	21.57 ± 1.07
INS (mIU/L)	68.04 ± 4.52	58.37 ± 2.77 *
IL -1β(ng/L)	43.37 ± 2.10	40.96 ± 2.82
IL -6 (ng/L)	1068.25 ± 111.94	1149.84 ± 110.57
IgA (µg/mL)	1683.52 ± 132.48	1617.45 ± 143.40
IgG (mg/mL)	54.74 ± 1.11	48.45 ± 4.05

UN = Urea nitrogen; GLU = Glucose; TP = total protein; ALB = Albumin; GLO = Globulin; TC = total cholesterol; ALP = Alkaline phosphatase; GH = Growth hormone; INS = Insulin; IL -1β = Interleukin -1β; IL -6 = Interleukin -6; IgA = Immunoglobulin A; IgG = Immunoglobulin G. For the same row, statistically significant differences between Ctrl and FLF groups were considered: * *p* < 0.05 and ** *p* < 0.01. Data are expressed as means ± SEM (n = 3).

**Table 8:** Effects of probiotic fermented liquid feed on apparent digestibility.

Items	Ctrl	FLF
OM (%)	86.29 ± 0.02	86.12 ± 0.06
CP (%)	72.78 ± 0.15	80.67 ± 0.28 **
EE (%)	83.01 ± 0.78	81.82 ± 1.02
NDF (%)	54.62 ± 1.23	54.66 ± 0.97
ADF (%)	48.05 ± 0.70	48.20 ± 0.77

OM = organic matter; CP = crude protein; EE = ether extract; NDF = neutral detergent fiber; ADF = acid detergent fiber. For the same row, statistically significant differences between Ctrl and FLF groups were considered: ** *p* < 0.01. Data are expressed as means ± SEM (n = 3).

**Table 9:** Effects of probiotic fermented liquid feed on meat quality.

Items		Ctrl	FLF
Drip force (%)		6.30 ± 0.32	4.15 ± 0.20 **
Shear force (N)		44.82 ± 0.92	41.72 ± 0.76 *
Cooking loss (%)		35.15 ± 1.05	28.50 ± 0.87 **
Flesh color	pH_45min_	6.64 ± 0.07	6.37 ± 0.19
	pH_24h_	5.55 ± 0.05	5.57 ± 0.02
	L* (lightness)	42.10 ± 0.65	41.62 ± 0.36
	a* (redness)	3.91 ± 0.16	4.26 ± 0.09 *
	b* (yellowness)	12.70 ± 0.27	11.01 ± 0.30 **

For the same row, statistically significant differences between Ctrl and FLF groups were considered: * *p* < 0.05 and ** *p* < 0.01. Data are expressed as means ± SEM (n = 3).

**Table 10:** Effects of fermented liquid feed on the small intestinal morphology of growing pigs.

Part	Items	Ctrl	FLF
Jejunum	Villus height (µm)	674.76 ± 55.01	1111.14 ± 60.54 **
	Crypt depth (µm)	426.01 ± 34.98	681.47 ± 54.61 **
	Villus: crypt	1.64 ± 0.27	1.64 ± 0.21
Ileum	Villus height (µm)	754.45 ± 43.35	966.04 ± 36.48 **
	Crypt depth (µm)	533.61 ± 53.76	469.81 ± 36.88
	Villus: crypt	1.43 ± 0.07	2.06 ± 0.13 **

For the same row, statistically significant differences between Ctrl and FLF groups were considered: ** *p* < 0.01. Data are expressed as means ± SEM (n = 3).

**Table 11:** Effects of complex probiotic fermentation on colonic volatile fatty acids.

Items	Ctrl	FLF
Acetic acid (mol/L)	55.32 ± 4.29	62.31 ± 0.64 *
Propionic acid (mol/L)	16.40 ± 0.82	19.29 ± 0.77 *
Butyric acid (mol/L)	8.28 ± 0.92	10.34 ± 0.34 *
Isobutyric acid (mol/L)	0.32 ± 0.0.05	0.64 ± 0.09 **
Isovaleric acid (mol/L)	0.89 ± 0.30	2.15 ± 0.23 **
Valeric acid (mol/L)	2.00 ± 0.46	1.64 ± 0.18

For the same row, statistically significant differences between Ctrl and FLF groups were considered: * *p* < 0.05 and ** *p* < 0.01. Data are expressed as means ± SEM (n = 3).

## Data Availability

None of the data were deposited in an official repository. The data are available from the authors upon request.
